# Effect of CAD–CAM Framework Design Fabricated from Sintered Cobalt–Chromium Alloy on Fracture Resistance of Metal–Ceramic Restorations

**DOI:** 10.1155/2023/3788590

**Published:** 2023-05-30

**Authors:** Kourosh Afshar, Mehran Falahchai, Homeira Ansarilari, Gelareh Tajziehchi

**Affiliations:** ^1^Department of Prosthodontics, Islamic Azad University Dental Branch, Tehran, Iran; ^2^Dental Sciences Research Center, Department of Prosthodontics, School of Dentistry, Guilan University of Medical Sciences, Rasht, Iran; ^3^Department of Restorative Dentistry, School of Dentistry, Guilan University of Medical Sciences, Rasht, Iran

## Abstract

**Introduction:**

Porcelain fracture is a common problem of metal–ceramic restorations (MCRs). One suggested strategy to prevent it is to modify the metal framework design; however, the available information regarding the effect of framework design on porcelain fracture is scarce.

**Objective:**

This study aimed to assess the effect of computer-aided design and computer-aided manufacturing (CAD–CAM) framework design fabricated from sintered cobalt–chromium (Co–Cr) alloy on fracture resistance of MCRs.

**Materials and Methods:**

Twenty premolar metal dies were fabricated for this in vitro study. Ten standard frameworks were designed with 0.5 mm thickness, and 10 customized frameworks were designed with 1 mm thickness at the lingual margin and 0.5 mm thickness in all other areas. All specimens were fabricated from sintered Co–Cr alloy (Ceramill Sintron) using soft metal milling technology. After porcelain application, the specimens underwent thermocycling and cyclic loading for 3,000 cycles between 5 and 55°C. The fracture resistance was measured by a universal testing machine. The failure mode was also determined. Data were statistically analyzed by independent *t*-test (*α* = 0.05).

**Results:**

The mean fracture resistance of porcelain was 2,379 ± 531 N in the standard and 2,557 ± 448 N in the customized group. No significant difference was found in fracture resistance of the two groups (*P* > 0.05). All specimens in both groups showed mixed failure.

**Conclusion:**

The fracture resistance of porcelain and the failure mode were not affected by the framework design of MCRs fabricated from sintered Co–Cr alloy (Ceramill Sintron).

## 1. Introduction

Metal–ceramic restorations (MCRs) are still widely used despite the advances in dental materials [1]. MCRs were the gold-standard prosthetic restorations in the past 40 years [2–4]. The metal framework of MCRs can be fabricated by the lost-wax technique, computer-aided design and computer-aided manufacturing (CAD–CAM) milling technology, or selective laser melting method [5, 6]. One significant advantage of the milling technique is that restorations are fabricated under standardized conditions. Thus, casting errors and porosities that can compromise the quality of restorations are largely prevented [7–9]. A high percentage of restoration problems occur due to impression and casting errors [9–11]. Also, the CAD–CAM milling technology has advantages such as lower dependence on the operator's skills, shorter working time, and higher precision due to fewer procedural steps, compared with the lost-wax technique. Thus, the CAD–CAM milling technology has gained increasing popularity [9, 11, 12].

Cobalt–chromium (Co–Cr) alloy is commonly used in clinical dental practice. The milled Co–Cr metal frameworks can be produced by hard metal milling (HMM) or soft metal milling (SMM) techniques. In the HMM technique, fewer errors occur compared with the casting method; however, the HMM technique is costly due to equipment wear as the result of metal hardness. The SMM of Co–Cr alloy is more cost-effective and less time-consuming than the HMM and casting techniques. In the SMM process, the metal is dry-milled and sintered at high temperatures to reach its full density with 10% volumetric shrinkage; therefore, after SMM, the Co–Cr alloy shows improved mechanical properties [13–15].

Chipping and fracture of porcelain are common and are regarded as the second most frequent cause of failure of MCRs after dental caries, with a prevalence rate of 2.3%–8% [16–19]. Trauma, inadequate occlusal adjustment, parafunctional habits, flexural fatigue, mismatch of the coefficients of thermal expansion (CTE) of metal and porcelain, inappropriate framework design, and errors in laboratory procedures are among the factors that may cause or contribute to chipping or fracture of porcelain [16, 20]. Several strategies have been suggested to minimize the rate of porcelain fracture, such as modifying the framework design [21].

The conventional framework design has 0.5 mm thickness in all areas. To improve the fracture resistance of restorations, the framework thickness in one commonly used modified design has increased to 1 mm at the lingual cervical margin, which increases to 2 mm and then continues to reach the proximal strut with 3.5 mm height. The thickness is 0.5 mm in all other areas [21, 22].

Several in vitro studies have evaluated the effect of CAD–CAM fabricated zirconia framework designs on fracture resistance [23–32]. Moreover, some studies evaluated the effect of framework design on fracture resistance of MCRs fabricated with the lost-wax technique [21, 22, 33]. However, to the best of the authors' knowledge, the effect of CAD–CAM framework design of MCRs fabricated from a soft metal alloy on porcelain fracture resistance has not been previously evaluated. Thus, this study aimed to assess the effect of CAD–CAM framework design fabricated from sintered Co–Cr alloy on fracture resistance of MCRs. The null hypothesis of the study was that there would be no significant difference between the fracture resistance of MCRs fabricated from sintered Co–Cr alloy (Ceramill Sintron) with standard and customized framework designs.

## 2. Materials and Methods

In this in vitro experimental study, the minimum sample size was calculated to be 10 in each group [34] assuming the statistical study power of 0.80, error rate of 0.05, standard deviations of 5.72 and 2.63 in the two groups, and *d* = 5.83, using the following Equation (1):

(1) $$\begin{array}{c} \begin{array}{c} n=\frac{{\left(z_{1-\frac{\alpha }{2}}+z_{1-\beta }\right)}^{2}\left(\sigma_{1}^{2}+\sigma_{2}^{2}\right)}{{\left(d\right)}^{2}}=\frac{{\left(1.96+0.84\right)}^{2}\left(5.72^{2}+2.63^{2}\right)}{{\left(5.83\right)}^{2}}=9.14≅10\\ d=\mu_{1}-\mu_{2}. \end{array} \end{array}$$

Twenty single-unit premolar MCRs were divided into two groups with customized framework design (*n* = 10) and standard framework design (*n* = 10) according to previous studies [19, 35]. Twenty maxillary second premolar acrylic teeth were collected and prepared with a deep-chamfer finish line with 1.2 mm depth and 8° occlusal taper of the walls along with 2 mm of occlusal reduction of the functional cusp and 1.5 mm of occlusal reduction of the nonfunctional cusp [21, 36]. The functional cusp was also beveled. The prepared acrylic tooth was scanned by using a desktop scanner (Ceramil Map400, Amann Girrbach AG, Koblach, Austria). The dies were carved out of wax discs (Yamahachi, Japan) by using a milling machine (Ceramil Motion 2, Amann Girrbach AG). The wax dies were then sprued and invested using phosphate-bonded investment (Z4 Universal Investment, N75, Belgium). The metal die was then cast (Duccatron Quattro, Ugin dentaire, France) with Co–Cr alloy (Magnum Ceramic Co., MESSA, Italy) [9, 37, 38]. The metal die was fixed on an acrylic stand and placed in a surveyor to make the necessary corrections in the preparation. Next, the metal die was scanned by a desktop scanner (Ceramil Map400; Amann Girrbach AG), and the data were transferred to CAD software (Ceramil mind CAD workstation; Amann Girrbach AG). The software program identified the finish lines, and the absence of undercuts was ensured [17, 39, 40].

**Figure 1:**
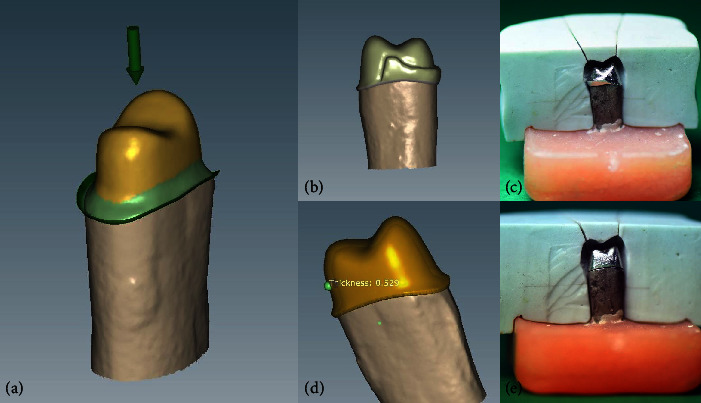
(a) Die spacer (50 *µ*) at 1 mm distance from the finish-line, (b) customized framework design, (c) prepared customized framework, (d) standard framework design, and (e) prepared standard framework.

Ten standard and ten customized frameworks were designed using the software (Ceramill Mind; Amann Girrbach AG). The thickness of the standard framework design was 0.5 mm in all areas. The customized framework design had 1 mm thickness in the lingual cervical margin, which increased to 2 mm and then continued to reach the proximal strut with 3.5 mm height. The thickness was 0.5 mm in all other areas [21, 22]. In both framework designs, the software considered 50 *µ*m space as the die spacer with 1 mm distance from the finish line (Figure 1). The sintered Co–Cr alloy blanks (Ceramill Sintron; Amann Girrbach AG) were dry-milled by using a communicating milling machine (Ceramil motion2; Amann Girrbach AG). The specimens were then sintered in a sintering furnace (Argovent, Ceramill Argotherm; Amann Girrbach AG) using inert argon gas at 1,280°C temperature for 6 hr. The fabricated specimens were all homogenous and did not have any distortion.  Table 1 shows the properties of the alloy used.

All specimens were visually inspected, and those with surface flaws or defects were replaced. Prior to veneering, the surface of the specimens was sandblasted (Basic eco microblaster, Renfort, Germany) with 50 *µ*m aluminum oxide particles from 10 mm distance at 45° angle for 20 s under 3 bar pressure according to the manufacturer's instructions. They were cleaned in an ultrasonic bath containing 80% ethanol for 5 min. The specimens were then placed in a furnace (Programat p310, Ivoclar Vivadent, Schann, Lichtenstein) for oxidation and degaussing.

**Figure 2:**
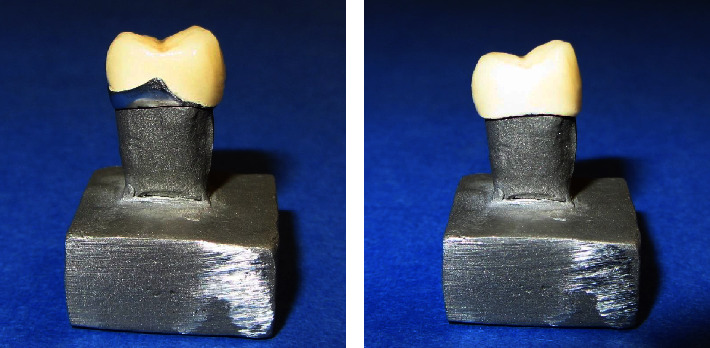
(a) Glazed customized framework, and (b) glazed standard framework.

The metal die was first scanned by a desktop scanner (Ceramil Map400; Amann Girrbach AG) and then a full-contour crown was designed by the Ceramill Mind design software. The wax pattern of the final crown was carved out of a wax disc (Yamahachi, Japan). The wax crown was placed on the die, and a putty index was obtained to standardize the porcelain application procedure. Two layers of opaque paste (Opaquer A3, Inline, Ivoclar Vivadent, Schann, Liechtenstein) were applied to all specimens. Then, the porcelain body (Schann, Liechtenstein) was applied to the specimens using the silicon index. The porcelain was finally glazed (Figure 2). The porcelain was sintered in a furnace (Programat p310, Ivoclar Vivadent, Schaan, Lichtenstein) according to the protocol described in Table 2. To prevent procedural errors, all the steps were carried out by a technician blinded to the study objectives. The CTE of porcelain was 12.9 ± 0.5 × 10–6 K, while the CTE of metal was 14.5 × 10–6 K.

Before cementation, the internal walls of the crowns and the metal dies were cleaned with alcohol and water steam. A thin layer of self-adhesive resin luting agent (RelyX Unicem, 3 M ESPE) was applied to the inner surface of the crowns. The restorations were then seated firmly on the die using finger pressure for 2 min. Then, they were maintained under 2.2 kg vertical force for 15 min and the excess cement was removed [38, 41]. One hour after cementation, the specimens were stored in water at 37°C for 1 week.

In order to simulate the oral clinical conditions, the specimens underwent thermocycling for 3,000 cycles between 5 and 55°C. Each cycle lasted for 60 s with a dwell time of 20 s and a transfer time of 20 s. This protocol simulated 2.5 years of clinical service [17, 39, 40].

**Figure 3:**
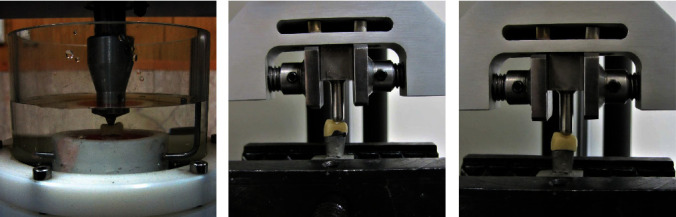
(a) Cyclic loading by applying 100,000 cycles of 100 N load with 1 Hz frequency corresponding to 2–3 months of clinical service, (b) load test of customized specimens, and (c) load test of standard specimens.

For cyclic loading, 20 metal dies were mounted in acrylic molds (Acropars acrylic resins, Marlic Co., Tehran, Iran), the crowns were seated, and the specimens were finally placed in a chewing simulator (CS4; SD, Mechatronik GMBH, Feldkirchen, Germany). The specimens were immersed in deionized water during the cyclic loading process [42]. Next, they underwent 100,000 cycles with 100 N load and 1 Hz frequency, corresponding to 2–3 months of clinical service (Figure 3(a)) [43].

They were placed in a tension/compression testing machine (Universal testing machine, DBBP 200, Santam, Tehran, Iran) with 200 KGFKGF capacity. The vertical load was applied by a stainless-steel round-end rod with 5 mm diameter parallel to the longitudinal axis at a crosshead speed of 1 mm/min until fracture [38]. To simulate contact with the opposing tooth, vertical load (N) was applied to the center of restorations. For this purpose, the load was applied to the triangular ridges of both facial and palatal cusps (Figures 3(b) and 3(c)) [44].

**Figure 4:**
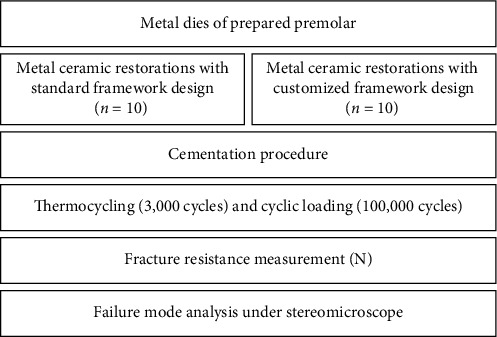
Flowchart of the experimental design of the present study.

The load-causing specimen fracture was recorded in Newtons (N). All specimens were inspected with the naked eye and also under a stereomicroscope (SMZ800, Nikon, Tokyo, Japan) at ×10 magnification to determine the mode of failure (as adhesive: between the alloy or ceramic and the oxide layer, cohesive: within the alloy or the ceramic material, or mixed: a combination of adhesive and cohesive) [7]. The flowchart of the present study design is shown in Figure 4.

Data were analyzed using SPSS version 24.0 (IBM Corp., Armonk, NY, USA). The Shapiro–Wilk test was used to assess the normality of data distribution, and Levene's test was applied to analyze the homogeneity of variances. The fracture resistance of the two groups was compared by the independent *t*-test. The level of statistical significance was set at 0.05.

## 3. Results

**Figure 5:**
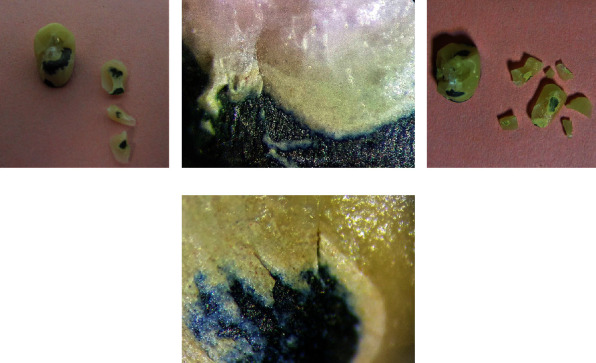
(a, b) Mixed failure of standard framework design, and (c, d) mixed failure of customized framework design.

As shown in Table 3, the mean and standard deviation of fracture resistance were 2,379 ± 531 N in the standard and 2,557 ± 448 N in the customized group. According to the independent *t*-test, this difference was not statistically significant (*P* > 0.05). As shown in Figure 5, all specimens in both groups showed mixed failure.

## 4. Discussion

This in vitro study assessed the effect of CAD–CAM framework design on porcelain fracture resistance of maxillary second premolar MCRs. The results revealed no significant difference in fracture resistance between the standard and customized framework designs. Thus, the null hypothesis of the study was accepted.

In vitro simulation of the clinical setting is often challenging. Fatigue is highly important in the clinical setting because restorations are subjected to different masticatory loads in the oral environment [45]. Moreover, water plays an essential role in the propagation of small cracks [7, 17, 46, 47]. Evidence shows that thermal and mechanical stresses adversely affect the metal–ceramic bond strength. Thus, specimens that have not undergone aging are expected to show higher bond strength and fracture resistance [48, 49]. Therefore, in the present study, the specimens underwent thermocycling and cyclic loading prior to the final testing of fracture resistance.

Using human teeth in such studies can lead to a more realistic simulation of the clinical setting [50]. However, human teeth are highly variable in terms of size, shape, and quality, and standardization of all specimens is not possible [51] Storage conditions and time passed since tooth extraction can also affect the fracture resistance and failure mode of the teeth [37]. Therefore, other materials such as metals, brass, epoxy resin, and composite resin have been suggested as alternatives; among which, it has been shown that composite resins are prone to premature fracture when using high-strength crowns [52]. The effect of the type of the supporting die on fracture resistance has been investigated in various types of all-ceramic restorations, reporting different results depending on the type of ceramic restoration [53, 54]. Sagsoz et al. [53] evaluated feldspathic monolithic ceramic crowns, and stated that metal and epoxy resin dies can be used in vitro. However, some other studies on all-ceramic crowns found that the elastic modulus of metal dies is much higher than that of human teeth and they deform less under forces, resulting in less stress accumulation in the inner surface of the crown [55]. They stated that the higher the elastic modulus of the substrate, the higher the reported fracture strength would be [51]. Therefore, when comparing the results of different studies, the type of the supporting die should be taken into account as an important parameter. However, the authors of the present study did not find any study on the effect of type of the supporting die on fracture resistance of MCRs. Many studies are available on MCRs with a metal die [22, 40, 56], which has an elastic modulus different from that of dentin. Therefore, the actual stress distribution in crowns cemented on human teeth is different from that in crowns cemented on a metal die [37]. In the present study, a metal die was used to standardize the preparation design of all substrates for the conduction of the loading test, but it may also be considered a limitation of the present study.

Also, deionized water was used as a lubricant in the process of cyclic loading in the present study. The specimens were thoroughly immersed in water. According to a previous study [42], since the type of liquid has no significant effect on the friction coefficient of natural teeth, deionized water was used instead of artificial saliva due to its availability and low cost.

According to the present results, the mean fracture resistance of porcelain was 2,379 ± 531 N in the standard and 2,557 ± 448 N in the customized group. Both framework designs could well tolerate the average bite force in the molar region (720 N) [57]. The maximum bite force can vary greatly. The average value in the molar region is 847 N in males and 597 N in females. The maximum bite force in patients with bruxism can exceed 800 N [58]. In another study, conducted on dentate young adults, the maximum bite force for individuals with bruxism was reported to be 806 N [59].

This study evaluated the MCRs fabricated from sintered Co–Cr alloy (Ceramill Sintron) by using the CAD–CAM technology, which is different from the methodology and materials used in studies conducted on MCRs fabricated by the lost-wax technique [21–23] or studies conducted on all-ceramic crowns with zirconia frameworks fabricated by using the CAD–CAM technology [19, 38, 60]. The present results revealed no significant difference in fracture resistance of porcelain in customized and standard framework designs fabricated from the sintered Co–Cr alloy (Ceramill Sintron) by using the CAD–CAM technology. This finding was in agreement with the results of Lorenzoni et al. [21] They demonstrated that the Ni–Cr framework design fabricated by the lost-wax technique had no significant effect on the fatigue life, although they did not perform thermocycling. In contrast, Bonfante et al. [22] and Bulbule and Motwani [33] showed that the Ni–Cr framework design fabricated by the lost-wax technique for maxillary premolars and maxillary central incisors significantly affected the fracture resistance of porcelain in MCRs [22, 33]. Unlike the present study, the abovementioned two studies did not perform cyclic loading or thermocycling for the aging of specimens. Differences in the type of alloy, framework design, type of tooth, and absence of aging simulation can explain the difference in the results. Moreover, previous studies on zirconia frameworks showed that the CAD–CAM framework design affected the fracture resistance of porcelain [19, 23, 24, 27, 38, 60]. Aside from the difference in framework material, different framework designs and absence of aging simulation in the abovementioned studies are the possible reasons for the variability in the results.

The mode of failure of MCRs can be adhesive (at the metal–porcelain interface), cohesive (within the metal or porcelain), or mixed (a mixture of adhesive fracture at the metal–porcelain interface and cohesive fracture within the ceramic or metal) [18]. In the present study, all fractures were mixed in both groups, which was in agreement with the results of Bonfante et al. [22] who reported that all fractures of porcelain fused to metal restorations caused coping exposure. Suleiman and von Steyern [7] reported that most specimens experienced a mixed fracture in their study. Cohesive fracture also occurred in some specimens, but no case of adhesive fracture was seen. The type, thickness, and properties of the cement used are among the influential factors on the failure mode of all-ceramic restorations, although conflicting results have been reported in this regard [61, 62]. It has been shown that the low elastic modulus of resin cement compared with glass ionomer cement results in the generation of flexural stresses during force application [62]. Furthermore, increasing the cement thickness can also cause deflection in the cement layer and generation of tensile stress in the crown surface [61]. In all-ceramic restorations, the internal surface is usually the main origin of fracture, especially in glass ceramics [63, 64]. Therefore, repeated tensile stresses at the core-cement interface can result in radial fractures, which are clinically manifested as bulk fractures [65, 66]. Despite the extensive research on metal–ceramic restorations, comprehensive characterization of their failure modes [21] and the effect of different parameters such as the type of cement in this respect are in need of further investigations.

In the current study, the failure of metal–ceramic restorations included actual fracture of the veneering porcelain; while, Lorenzoni et al. [21] demonstrated field damage by creating internal and external cone cracks without actual fracture of the veneering porcelain. Therefore, future studies are recommended to assess the core–cement interface. However, it has been reported that in studies that use spherical indents (such as the present study), the resulting cone cracks are more related to high stresses at the contact surface rather than tensile stresses at the cement–crown interface [67].

Despite all considerations, the present study had an in vitro design, and therefore could not perfectly simulate the clinical oral conditions. Similar to many other in vitro studies, the specimens were only subjected to vertical loads in the present study. In the oral environment, however, the MCRs are subjected to loads applied from different directions. This difference is a limitation of the present study that limits the generalizability of the results to the clinical setting. Further studies are recommended to assess and compare the effect of different alloys, die materials, cements, framework designs, and fabrication methods on fracture resistance of MCRs.

## 5. Conclusion

Considering the limitations of this study, the standard and customized sintered Co–Cr alloy (Ceramill Sintron) framework designs fabricated by using the CAD–CAM technology had no significant effect on fracture resistance of porcelain in MCRs. Also, the porcelain failure mode was mixed in all specimens, irrespective of the framework design.

## Figures and Tables

**Table 1:** Physical and chemical properties of the alloy used in this study.

Ingredients	Cobalt (Co)	Chromium (Cr)	Molybdenum (Mo)	Tungsten (W)	Others (Si, Fe, Mn)
	66%	28%	5%	–	∼1%
Yield strength	450 MPa				
Modulus of elasticity	200 MPa				
Elongation at fracture	30%				
Vickers hardness (HV 10)	270 HV 10				
Coefficient of thermal expansion (25–500°C)	14.5 × 10^−6^ k^−1^				
Density	7.9 g/cm^3^				

**Table 2:** Porcelain sintering protocol according to the manufacturer (IPS Inline Ivoclar).

	*T* (°C/°F)	*B* (°C/°F)	*S* (min)	*t* ^↗^ (°C/°F/min)	*H* (min)	*V*_1_ (°C/°F)	*V*_2_ (°C/°F)
1^st^ and 2^nd^ paste opaquer	930/1,706	403/757	6	100/180	2	450/842	929/1,704
1^st^ dentin	910/1,670	403/757	4	60/108	1	450/842	909/1,668
2^nd^ dentin	900/1,652	403/757	4	60/108	1	450/842	899/1,650
Glaze	850/1,562	403/757	6	60/108	2	450/842	849/1,560

**Table 3:** Fracture resistance of the standard and customized framework designs in Newtons (N).

	Standard (*n* = 10); mean ± SD	Customized (*n* = 10); mean ± SD
Fracture resistance	2,379 ± 531	2,557 ± 448
*P*-value	0.602	

SD, standard deviation.

## Data Availability

The data used to support the findings of this study will be available from the corresponding author upon request.
